# Phylogeny-aware linear B-cell epitope predictor detects targets associated with immune response to orthopoxviruses

**DOI:** 10.1093/bib/bbae527

**Published:** 2024-11-06

**Authors:** Felipe Campelo, Ana Laura Grossi de Oliveira, João Reis-Cunha, Vanessa Gomes Fraga, Pedro Henrique Bastos, Jodie Ashford, Anikó Ekárt, Talita Emile Ribeiro Adelino, Marcos Vinicius Ferreira Silva, Felipe Campos de Melo Iani, Augusto César Parreiras de Jesus, Daniella Castanheira Bartholomeu, Giliane de Souza Trindade, Ricardo Toshio Fujiwara, Lilian Lacerda Bueno, Francisco Pereira Lobo

**Affiliations:** School of Engineering Mathematics and Technology, University of Bristol, Ada Lovelace Building, Tankard's Close BS8 1TW, Bristol, United Kingdom; Post-Graduate Program in Infectious Diseases and Tropical Medicine, School of Medicine, Federal University of Minas Gerais, Av. Prof. Alfredo Balena 190, 30130-100, Belo Horizonte, Brazil; York Biomedical Research Institute, Department of Biology, University of York, Wentworth Way YO10 5NG, York, United Kingdom; Department of Parasitology, Institute of Biological Sciences, Universidade Federal de Minas Gerais, Av. Pres. Antônio Carlos, 6627, 31270-901, Belo Horizonte, Brazil; Department of Microbiology, Institute of Biological Sciences, Universidade Federal de Minas Gerais, Av. Pres. Antônio Carlos, 6627, 31270-901, Belo Horizonte, Brazil; Immigrant and Global Health, Global Tuberculosis Program, Department of Pediatrics, Baylor College of Medicine, 1 Baylor Plz, Houston, TX 77030, United States; Aston Centre for Artificial Intelligence Research and Application, Aston University, Aston Triangle B4 7ET, Birmingham, United Kingdom; Aston Centre for Artificial Intelligence Research and Application, Aston University, Aston Triangle B4 7ET, Birmingham, United Kingdom; Central Public Health Laboratory, Fundação Ezequiel Dias, R. Conde Pereira Carneiro, 80, 30510-010, Belo Horizonte, Brazil; Central Public Health Laboratory, Fundação Ezequiel Dias, R. Conde Pereira Carneiro, 80, 30510-010, Belo Horizonte, Brazil; Central Public Health Laboratory, Fundação Ezequiel Dias, R. Conde Pereira Carneiro, 80, 30510-010, Belo Horizonte, Brazil; Post-Graduate Program in Infectious Diseases and Tropical Medicine, School of Medicine, Federal University of Minas Gerais, Av. Prof. Alfredo Balena 190, 30130-100, Belo Horizonte, Brazil; Department of Parasitology, Institute of Biological Sciences, Universidade Federal de Minas Gerais, Av. Pres. Antônio Carlos, 6627, 31270-901, Belo Horizonte, Brazil; Department of Microbiology, Institute of Biological Sciences, Universidade Federal de Minas Gerais, Av. Pres. Antônio Carlos, 6627, 31270-901, Belo Horizonte, Brazil; Department of Parasitology, Institute of Biological Sciences, Universidade Federal de Minas Gerais, Av. Pres. Antônio Carlos, 6627, 31270-901, Belo Horizonte, Brazil; Department of Parasitology, Institute of Biological Sciences, Universidade Federal de Minas Gerais, Av. Pres. Antônio Carlos, 6627, 31270-901, Belo Horizonte, Brazil; Department of Genetics, Ecology and Evolution, Institute of Biological Sciences, Universidade Federal de Minas Gerais, Av. Pres. Antônio Carlos, 6627, 31270-901, Belo Horizonte, Brazil

**Keywords:** epitope prediction, phylogeny-aware methods, machine learning, orthopoxvirus, monkeypox virus, diagnostics

## Abstract

We introduce a phylogeny-aware framework for predicting linear B-cell epitope (LBCE)-containing regions within proteins. Our approach leverages evolutionary information by using a taxonomic scaffold to build models trained on hierarchically structured data. The resulting models present performance equivalent or superior to generalist methods, despite using simpler features and a fraction of the data volume required by current state-of-the-art predictors. This allows the utilization of available data for major pathogen lineages to facilitate the prediction of LBCEs for emerging infectious agents. We demonstrate the efficacy of our approach by predicting new LBCEs in the monkeypox (MPXV) and vaccinia viruses. Experimental validation of selected targets using sera from infected patients confirms the presence of LBCEs, including candidates for the differential serodiagnosis of recent MPXV infections. These results point to the use of phylogeny-aware predictors as a useful strategy to facilitate the targeted development of immunodiagnostic tools.

## Introduction

B cells constitute a major component of vertebrate adaptive immunity. These cells rely on B-cell receptors to interact with epitopes, defined as smaller regions within antigens recognized by the host immune system and, in the case of B cells, capable of stimulating antibody production. B-cell epitopes are commonly classified as either linear (continuous) or conformational (discontinuous) [1]. The computational prediction of linear B-cell epitopes (from now on referred to as LBCEs) has been under active development for 40 years [2] and has become a crucial step in the fast development of vaccines and diagnostic tests for infectious diseases [3].

A common premise in this field is the development of generalist predictors, defined as models trained with a taxonomically heterogeneous set of entries and intended to generalize to any LBCE prediction task, irrespective of the pathogen being studied. However, biological characteristics are not independent across lineages due to common ancestry, as species sharing a more recent common ancestor are expected to be more similar in both their phenotypes and genotypes [4]. Recently, our group demonstrated how organism-specific training generates consistent improvements over generalist methods for LBCE prediction [5]. This suggests that it is possible to deploy tailored models that incorporate phylogenetic information to optimize predictive performance for specific groups of pathogens [6]. Importantly, these models can be trained using data from well-characterized groups of pathogens and generalized to related data-poor species, such as neglected infectious diseases and emerging and reemerging human pathogens.

The 2022 and 2024 outbreaks of the monkeypox virus (MPXV) represent recent examples of an emerging data-poor pathogen of global concern. MPXV is a member of the vertebrate-infecting branch of the Poxviridae family (orthopoxviruses, from now on referred to as OPVs) [4]. In humans, OPVs include the variola virus (VARV), the infectious agent of smallpox, one of the most devastating epidemics in history. OPVs also include viruses causing zoonotic diseases, such as the cowpox virus (CPXV) and the vaccinia virus (VACV); the latter is also the source of the modern smallpox vaccine. Even though MPXV outbreaks had been previously reported in Africa, Asia, North America, and Europe; the recent ones have been the largest and most concerning ones, comprising 99 176 officially recorded cases in 116 countries and territories across the five World Health Organization (WHO) regions between 1 January 2022 and 30 June 2024, with 208 recorded deaths according to the most recent WHO report on the multicountry outbreak of Mpox [5]. At the time of the outbreak and up to early 2024, only five MPXV LBCE-containing regions were available in the Immune Epitopes Database (IEDB) [6], with no validated counterexamples.

In this study, we present a novel phylogeny-aware modeling framework that relies on a taxonomic scaffold to build bespoke models for detecting LBCE-containing regions. This strategy is deployed to develop a predictor optimised for orthopoxviruses, which we deploy to detect targets in MPXV and VACV proteins. By employing a data-filtering strategy based on evolutionary relationships between the source organisms of known epitopes/nonepitope peptides and the target pathogen, we achieve results that are comparable to, if not better than, the state-of-art tools, despite using a much smaller and computationally simpler feature space combined with a traditional data mining workflow. The resulting model has high predictive performance in detecting LBCE-containing regions, which is validated both *in silico* and experimentally. Nine selected targets were validated on sera from cohorts of patients known to have been infected with either MPXV or VACV, confirming that all nine contain LBCEs of one or both viruses and highlighting peptides of potential diagnostic value.

## Materials and methods

### Ethics statement

The use of human serum in this study was approved by the Human Research Ethics Committees of Fundação Ezequiel Dias (Process number (CAAE): 62702222.6.0000.9507—MPXV samples and some VACV samples) and of the Federal University of Minas Gerais (CAAE: 42277020.5.0000.5149—additional VACV samples). All participants consented by signing an informed consent form before their involvement in the study. For participants under the age of 18, parents or legal guardians provided consent by signing the informed consent form. Full details on the samples and ethical approvals are available upon request.

### Sequence data sets

**Figure 1:**
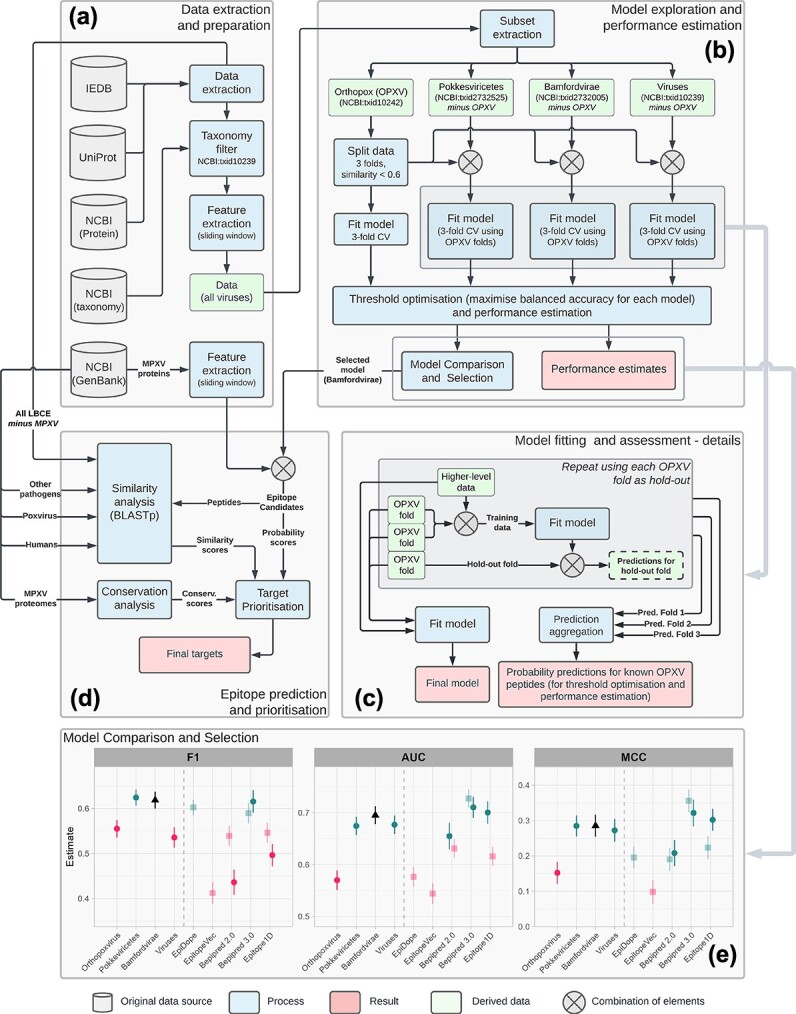
Computational pipeline for the development and selection of OPXV-specific models and the prediction and prioritization of candidate monkeypox LBCE-containing regions. (a) Data sources and data set generation process. Queries to the IEDB, UniProt, and NCBI databases were automated using the development version of R package *epitopes* [7]. The MPXV proteins were retrieved from the Genbank file corresponding to the first complete genome sequence of the 2022 MPXV outbreak (isolate MPXV USA 2022 MA001, accession number ON563414). (b) Model development and assessment. The detailed view of the model training process (c) highlights the use of OPXV folds for model training (combined with data from higher taxonomic levels) and performance assessment. In this work, all model fitting was done using RFs, following the preliminary modeling explorations reported in our earlier work [10]. (d) Use of the selected model (retrained with all OPXV examples after the performance assessment, as shown in block c) to predict epitopes on MPXV proteins. The candidate peptides were ranked based on predicted probability, conservation in MPXV, low similarity to known epitope-containing regions from other pathogens, and low alignment scores to proteins from other viruses and from the human proteome. (e) Performance assessment and comparison. The performance of the selected model (“Bamfordvirae”) is compared against other models trained with data filtered at different taxonomic levels (left side of each panel) as well as against predictors from the literature (right side of each panel). Statistically significant differences are highlighted in red, whereas differences that are not significant at the 95% FDR-corrected confidence level are shown in dark green (this information is also provided in a color-neutral manner in Supplementary Table ST4). Vertical bars represent standard errors of estimation. Lighter-shaded estimates with square markers indicate the performance of models subject to data leakage (see the Results section and Supplementary Table ST4). All models used thresholds optimized to maximize balanced accuracy on the OPXV data, aiming at generating a good balance between sensitivity and specificity.

The process of retrieving and processing the relevant sequence data sets is illustrated in Fig. 1a. Data extraction, filtering, and consolidation were performed using functions available in the development version of R package ‘epitopes’ [7], based on the full XML export of IEDB [6] on 20 May 2022. Taxonomy information was retrieved from the National Center for Biotechnology Information (NCBI)'s taxonomy dataset. All entries identified as LBCEs from organisms under the superkingdom Viruses (NCBI:txid10239) were extracted from the IEDB export, with associated proteins retrieved from the NCBI protein database [8] and UniprotKB [9]. Peptides were labeled as positive if half or more of the assays associated with that IEDB entry reported a positive result, and positive-labeled peptides of length >30 amino acid residues were removed, to prevent long “epitope-containing regions” from adding excessive noise to the training data. Overlapping peptides of the same class were merged into a single entry to prevent partial data duplication. The resulting sequence data set was tabularized using a sliding window strategy (window length = 15, step size = 1) [10], and a set of 385 statistical and physicochemical features (Supplementary File 1) based on the local window around each residue were calculated.

The resulting data set containing information on all labeled peptides related to viral LBCEs was further divided as follows. All entries related to pathogens under the genus orthopoxvirus (OPXV) (NCBI:txid10242) were isolated in a separate data set, and a similarity-aware data-splitting strategy was used to split the data into three folds based on normalized local alignment scores, to prevent data leakage due to protein homology (Supplementary File 1). Four of the positive examples available under the OPXV data correspond to known MPXV epitopes (IEDB epitope IDs 10135, 7309, 39258, and 73976), with no recorded negative MPXV examples.

The remaining entries (excluding all OPXV ones) were used to generate three data sets, with all entries related to pathogens under (i) class Pokkesviricetes (NCBI:txid2732525); (ii) kingdom Bamfordvirae (NCBI:txid2732005); and (iii) the Viruses superkingdom (NCBI:txid10239). Supplementary Table ST1 summarizes the main characteristics of these data sets.

The predicted proteome corresponding to annotation data related to the isolate MPXV_USA_2022_MA001 (Genbank—ON563414) was retrieved from NCBI. In total, 190 proteins were retrieved and processed similarly to the training data. We also gathered genomic data from MPXV isolates before the outbreak (all complete genomic sequences available as of June 2022, 88 samples) and from VACV isolates (all complete genomic sequences available as of June 2022, 68 samples) to evaluate sequence conservation.

### Predictive modeling

The training and assessment of models for LBCE prediction in pathogens under the genus Orthopoxvirus are detailed in Fig. 1. Pathogen-specific training improves the performance of LBCE predictors when compared to generalist models trained with larger but considerably more taxonomically heterogeneous data [10]. As the number of available MPXV-specific examples in IEDB is very low to train—or even properly assess—any predictive models, we used the same rationale presented by Ashford *et al*. [10] to investigate models that generalize well to our genus of interest, i.e. models displaying the good predictive ability to detect orthopoxvirus LBCE.

Random Forest (RF) models were trained using the data sets described in subsection Sequence Data Sets. These datasets present increasing amounts of data, obtained by expanding the inclusion criterion to admit viral lineages with increasing taxonomic distance from OPXV. The model trained using only the OPXV data was assessed using 3-fold cross-validation with similarity-based folds as described in subsection Sequence Data Sets. The models trained with data from higher taxonomic levels were also assessed using 3-fold cross-validation, with each submodel trained on the combination of the full higher-level data set plus two folds of the OPXV data and deployed to predict the examples in the third (holdout) OPXV fold. These predictions were then aggregated together for threshold optimization and performance comparison. After the predicted residue-wise probabilities for each hold-out fold were aggregated and recorded, a final model was then generated for each of the datasets consisting of the full OPXV data combined with one set of higher-level observations (Pokkesviricetes, Bamfordvirae, and Viruses, respectively). This process is detailed in Fig. 1c.

The vectors of predicted probabilities for each residue of the OPXV data were used to optimize the classification threshold for each model. This process was done by a simple iterative search, varying the threshold from 0*.*01 to 0*.*99 in increments of 0*.*01 and calculating the balanced accuracy (the simple mean of model specificity and sensitivity) at each level. The threshold values that resulted in the highest balanced accuracy were selected for each model.

All RF models were trained using the implementation from R package *ranger* [11] version 0.13.1 with hyperparameters as detailed in Supplementary File 1. Class imbalance was treated by stratified undersampling of the majority class, ensuring that all peptides remained represented in the rebalanced data.

The resulting RF classifiers can be applied to predict the LBCE probability for each residue of a given protein and return a probability value for each protein position. In this work, contiguous sequences of at least eight residues above the optimized model threshold were considered as potential epitope-containing peptides, with the probability score of the peptide attributed as the mean of the predicted epitope probabilities of its amino acid residues.

### Performance assessment and comparison

The performance obtained by the models trained on each data set for the prediction of OPXV epitopes was quantified using commonly used performance indicators. Six of those represented aggregate performance indices: area under the ROC curve (AUC), F1 indicator (F1), Matthews correlation coefficient (MCC), accuracy, balanced accuracy, and geometric mean of sensitivity and specificity (G-mean); whereas another four quantified specific aspects of predictive performance: sensitivity, specificity, precision (positive predictive value, PPV) and negative predictive value (NPV) [12]. Standard errors for each value and *P*-values for individual comparisons of the selected model against the other tested models were estimated using bootstrap. We tested four generalist LBCE predictors as baselines for comparison: Bepipred 2.0 [13] (the standard LBCE prediction tool provided by the IEDB [6]), Bepipred 3.0 [14], EpiDope [15], and EpitopeVec [16]. We also included a recent taxon-specific LBCE predictor, the Varidnaviria model provided by Epitope1D [17], to contrast the performance of our models with another taxon-specific approach. Details of the bootstrap methodology used to estimate confidence intervals and *P*-values, as well as of the correction for multiple hypothesis testing, are provided in Supplementary File 1.

### Study population and plasma samples

Three sera panels were used to evaluate the antigenicity of the selected peptides. The first sera panel corresponds to 47 MPXV-positive individuals aged 18–54 years old, obtained from the Central Laboratory of Public Health of Minas Gerais (*Laboratório Central de Saúde Pública do Estado de Minas Gerais*), where they were sent for the molecular diagnosis of MPXV. All the samples were collected in 2022. The second sera panel corresponds to 83 VACV-positive patients. These samples were obtained by the UFMG Virus Laboratory from *Secretaria de Saúde do Estado de Minas Gerais*, the health department under the government of Brazilian state of Minas Gerais, as well as from previous seroprevalence studies performed by the laboratory [18, 19]. The samples were collected between 2015 and 2021 from different municipalities and regions with endemic VACV circulation in the state of Minas Gerais, Brazil. The age of the individuals ranged from 6 to 88 years at the time of collection, and 66% (55/83) of the individuals had received the antivariolic vaccine. Finally, the third sera panel corresponds to healthy human donors, obtained from adult volunteers from nonendemic area of Minas Gerais State, Brazil.

### Experimental validation of the predicted linear B-cell epitopes

A total of 179 peptides of 15 amino acids, representing overlapping portions that cover the full extent of the predicted targets, were synthesized in duplicate in a peptide-array using the automated peptide synthesizer ResPep SL (Intavis®) through SPOT-Synthesis technique [20] on amino-PEG (polyethylene glycol) cellulose membrane. The IgM and IgG reactivities to each peptide was assessed using pools of sera from patients infected with monkeypox virus (MPXV—12 patients), vaccinia virus (VACV—12 patients), or healthy donors (negative control—10 individuals) by Immunoblotting (Supplementary File 9). The selection of sera to be used in the pools was by random sampling. The pools of sera were diluted 1:1000 in PBS Tween 0.1% and incubated for 2 h with the membrane and washed. Then, the secondary antibodies (IgM or IgG), diluted 1:20 000–1:25 000 in PBS Tween 0.1, were incubated with the membrane for 1 h and washed. The reactive spots were visualized with chemiluminescence (Supplementary File 9).

Reactivity values for each peptide were determined by densitometry, using the ImageJ software and the Protein Array Analyzer plugin [21], subtracting from each spot the mean value of four negative controls. The positivity cutoff was established as twice the reactivity from the immunoblotting assay using the pool of sera from healthy donors. Finally, the reactive intensities from each peptide and each pool of sera were compared, to identify potential MPXV peptides and controls. Details of the immunoblotting method are provided in Supplementary File 9.

The soluble peptide (DVKVEEKNIIDIEDD) was synthesized on a 10 μmol scale using the ResPep SL automated synthesizer (Intavis®) and confirmed by mass spectrometry using Autoflex Speed MALDI/TOF equipment (Bruker). Details of the peptide synthesizing method, as well as of the enzyme-linked immunosorbent assay (ELISA) used to assess the reactivity of this peptide to individual patient sera, are provided in the Supplementary File 9.

## Results

### Phylogeny-aware modeling yields superior predictive performance for orthopoxvirus

The data mining pipeline developed in this work (Fig. 1) consists of a set of R protocols built around the ‘epitopes’ package [7], which uses machine learning (ML) to predict LBCE-containing regions optimized for specific target pathogens. Notably, our strategy also incorporates phylogenetic information, allowing the production of hierarchical models by iterating through a phylogenetic tree to produce models for increasingly diverse groups of phylogenetically related pathogens. The development of this pipeline was motivated by earlier results showing that organism-specific training of epitope predictors resulted in substantial gains in predictive performance over generalist models [10], even when the total amount of training data was substantially reduced [22]. By iterating through different taxonomic levels, it is possible to gather more training examples from phylogenetically related organisms. Since these are also expected to be phenotypically and genetically more similar, these data entries may be used to both increase the taxonomic coverage of models and the number of training data points, as well as capturing common features of viral lineages due to common ancestry. One important exception is the model trained on the “Viruses” dataset: since the full set of viral lineages is not monophyletic [23], a model trained using this dataset is expected to have a performance equivalent to other generalist approaches trained on vast amounts of epitope data from diverse pathogens.

The pipeline consists of a data extraction and preparation module (Fig. 1a), which includes extracting and consolidating LBCE data and calculating a feature space composed of 385 statistical and physicochemical descriptors that have been widely adopted in earlier approaches to predict LBCEs [10] (Supplementary File 1), and a model exploration and assessment module (Fig. 1b), in which RF classifiers are trained on data from progressively higher taxonomic levels (Supplementary Table ST1) and assessed on data from OPXV using a bespoke cross-validation approach (described in section Predictive Modeling and detailed in Fig. 1c and Supplementary File 1, “Data Splitting for Cross Validation”). The classification threshold for each model was selected as the one that maximized balanced accuracy, to adequately balance the resulting pipeline’s sensitivity and specificity.

The predictive performance of models trained on data from different taxonomic levels showed a clear increase across all aggregated performance metrics as more data from phylogenetically related pathogens sharing a more ancient common ancestor was added to the training set (Fig. 1e and Supplementary Table ST4). The lower performance of the model trained exclusively with the OPXV data (or, more precisely, the average performance of models trained with two-thirds of the available OPXV data on IEDB; see Fig. 1c) is likely due to the extremely low sample size available at this level, which is likely insufficient to fit models with good generalization ability, as indicated previously [22]. The performance of our models stabilized at the taxon Bamfordvirae, with a decrease of some performance metrics in the more general model trained with all viral sequences (Fig. 1e and Supplementary Table ST4). This result reinforces observations reported in our earlier study [10], which indicated that training models using data related to the target pathogen results in gains of predictive ability due to the fact that an increase in the amount of data from phylogenetically close species are available to train the models, while neutral or even negative effects on performance are expected when the training data are obtained from more distantly related pathogens or nonmonophyletic lineages, as is the case of the “Viruses” dataset in our analysis [23], even when the total data volume is substantially increased.

We compared the performance of our models against four generalist predictors trained using phylogenetically diverse sets of positive and negative examples that are considerably greater than our training set, as well as against a taxon-specific tool (see Fig. 1e; Supplementary Table ST4). Three of the generalist tools are arguably the state-of-the-art software in terms of feature modeling and ML strategies for LBCE prediction: EpiDope [15], EpitopeVec [16], and Bepipred 3.0 [14]. These state-of-art generalist tools adopt protein language models to extract numerical representations of amino acid features as sequence embeddings, to capture both local and context-dependent properties. This representation schema has been demonstrated to provide good performance in many ML strategies for the analysis of protein sequences, such as prediction of protein structure, subcellular location, and biological function [24–26]. In particular, Bepipred 3.0 adopts the Evolutionary Scale Modelling 2 model, a cutting-edge protein language model initially applied to protein structure prediction [27]. Two of these tools (Bepipred 3.0 and EpiDope) employ deep learning to develop their classifiers, which has also been widely adopted for mining protein data due to its often-superior performance compared to traditional ML methods [28]. These methods therefore present more sophisticated feature spaces and ML strategies when compared to our approach. We also include the previous version of Bepipred (version 2.0) [13], as this is currently the main available tool for LBCE prediction provided by IEDB. This tool is also the most similar to our strategy in terms of feature space and learning method for classifier building, therefore providing a useful benchmark to compare how our phylogeny-aware modeling strategy performs against a conceptually similar but generalist LBCE prediction tool. Finally, we compared the performance of our selected model against a taxon-specific model developed specifically for Varidnaviria, a clade that includes the Bamfordvirae kingdom. This model, which is one of the taxon-specific predictors provided by Epitope1D [17], employs a graph-based feature extraction step based on statistical and physicochemical properties and Explainable Boosting Machine predictors and was reported as providing taxon-specific performance comparable or superior to that of Bepipred 3.0, EpitopeVec, and EpiDope [17].

Data leakage due to sequence similarity may be a major issue when evaluating the performance of classifiers of biological sequences [29]. To quantify the influence of data leakage due to sequence similarity, we extracted two sets of sequences to assess performance for each of the methods evaluated. The first set includes all OPXV entries, including sequences that are highly similar to entries used to train the four models from the literature (Fig. 1e, transparent colors with square markers). This data s*et* allows data leakage due to sequence similarity, since some of the entries used to train these tools correspond to entries in the validation data set. It may therefore result in positively biased performance estimates for the methods affected by the leakage. We used BLASTp with the same configuration previously described to select small sequences followed by a stringent filter (query coverage >80% and identity of the aligned region >80%) to produce a second, “no-leakage,” validation data set for each method (Fig. 1e, solid colors). This set is expected to enable the estimation of the true generalization performance of these classifiers, as it only contains sequences that are not found in their training data sets. No-leakage performance estimation was not possible for EpitopeVec (since all OPXV entries used in this study have correspondence in its training set) and EpiDope (all but six entries, all positive). Therefore, we report only the results with data leakage for these two tools.

From the models trained using our phylogeny-aware strategy, the one trained using data at the Bamfordvirae level was found to return the best overall combination of performance scores (Fig. 1e, Supplementary Table ST4). We selected this model and used it to compare the relative classifier performance of the other tools using all-versus-one comparisons. P-values were corrected for multiple hypotheses testing to maintain a predefined false discovery rate (FDR) of 5% (see Materials and Methods, subsection Performance Assessment and Comparison; and Supplementary File 1). The performance of our selected model is either indistinct or significantly better than all models tested regardless of data leakage. This is despite the use of a considerably smaller training set and our adoption of straightforward features and a basic ML strategy when compared to the state-of-art models. Of note, our best model significantly outperforms EpitopeVec (leaky), Bepipred 2.0 (leaky and no-leakage), and Epitope1D (leaky and no-leakage) in terms of the F1-score and EpiDope*, EpitopeVec (leaky), Bepipred 2.0 (leaky), and Epitope1D (leaky) for AUC and is statistically indistinguishable from Bepipred 3.0 (leaky and no-leakage) across all three metrics and from Epitope1D (leaky and no-leakage) for MCC. Similar patterns are observed across several other performance indicators (Supplementary Table ST4). Taken together, these results strongly suggest that the phylogeny-aware modeling strategy is the main factor behind the surprisingly high performance of our best model compared to the many instances of generalist models evaluated. This is further reinforced by the similar performance between our best model and the Epitope1D (Varidnaviria) model, which was trained using a similar phylogeny-aware data filtering approach. Based on these results, the model trained at the Bamfordvirae level was selected for downstream analyses.

### Orthopoxvirus-optimized model predicts new linear B-cell epitope candidates for monkeypox virus

We used the genome of MPXV as a scaffold to anchor our prediction results. For that, we deployed the selected model (trained on all available examples under kingdom Bamfordvirae and optimized to maximize balanced accuracy on OPXV data) to generate predictions for the 190 proteins derived from the Genbank file corresponding to the first complete genome sequence of the 2022 MPXV outbreak (isolate MPXV USA 2022 MA001, accession number ON563414). Supplementary Files 2 and 3 provide the details of the 190 proteins and a summary of the number of peptides predicted as epitope-containing regions, as well as the maximum peptide probability found for each protein entry.

### Conservation and similarity analysis suggest potential linear B-cell epitopes for diagnostic tests

**Figure 2:**
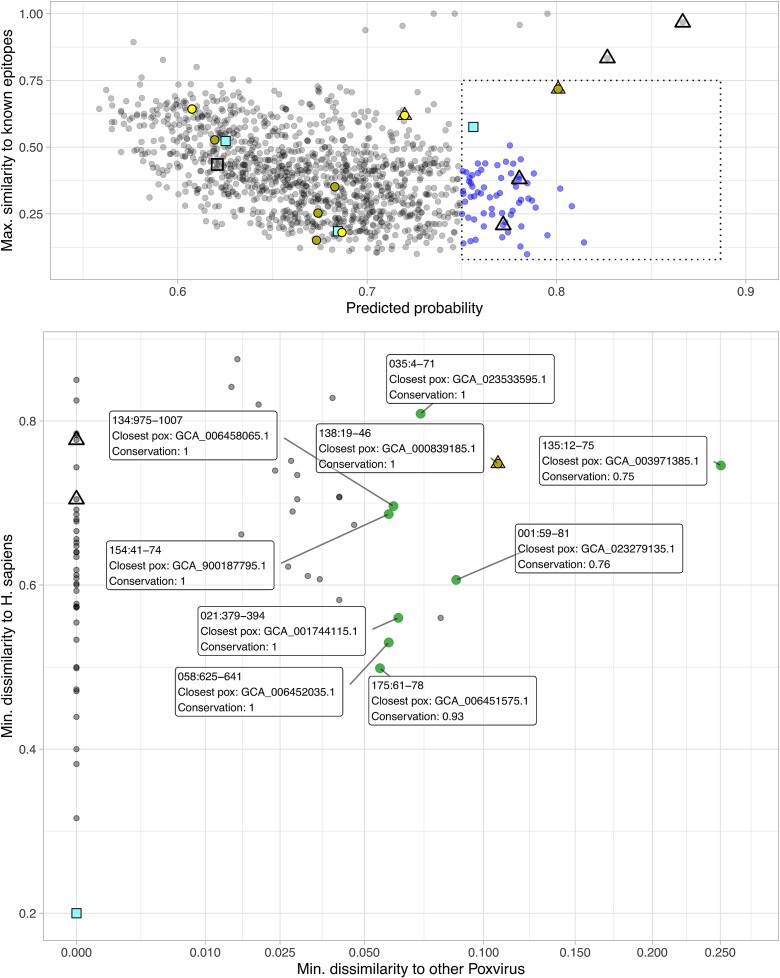
(Top) Predicted epitopes in the space of predicted probability × max. similarity to known LBCE. Peptides with low similarity and high predicted probability were highlighted and selected for further analysis. These points were projected onto the space of minimum dissimilarity to proteins from other poxvirus × *H. sapiens* (bottom). Several peptides with perfect or near-perfect similarity with other poxvirus (shown on the left of the bottom plot) would be unsuitable for the development of diagnostic tools to differentiate MPXV infections from other poxviruses. Predicted LBCE with a conservation score ≥0.75, dissimilarity to human proteins ≥0.5 and dissimilarity to other poxvirus proteins ≥0.05 are labeled in the bottom panel and were selected for experimental assessment as potential candidates for diagnostic test development. Predicted peptides that match MPXV epitopes available from IEDB are shown as triangles (filled in the case of “new” IEDB epitopes, not available at the time of model training); those that match the epitope-containing regions described by Yates *et al*. [30] are highlighted as squares (filled for targets selected in Yates *et al*.’s work to compose their assay); and those that match the ones used by Taha *et al*. for the development of an MPXV serological assay [31] are shown as circles (a brighter shade indicates peptides chosen to compose the diagnostic test in that work).

To evaluate the prediction capabilities of our tool and identify potential diagnostic targets capable of differentiating between MPXV and other infections, the model predictions were filtered according to additional criteria. The selected model predicted 1241 unique peptides with probabilities above the optimized model threshold (Fig. 2, top). Our model successfully identified all known MPXV epitopes from IEDB (triangle markers), including “new” IEDB epitopes, not available at the time of model training (red triangles). Our model also predicted peptides that match the epitope-containing regions described in [30] (square markers), including the targets selected by Yates *et al.* to compose their diagnosis test (cyan squares).

Initially, 66 predicted LBCE-containing regions with model probability >0.75 and similarity scores (calculated as coverage × identity) to known viral LBCE-containing regions extracted from IEDB lower than 0.75 (to minimize the probability of cross-reactivity) were selected for further analysis. As a second filter, we assessed the sequence conservation of these peptides among 88 MPXV isolates as available in Genbank in June 2022 and dissimilarity to proteins from the human host and other viruses. We estimated the conservation of the candidate peptides for the predicted proteome of MPXV isolates using BLASTp [32] (options -seg no e-value 10000 -word size 3, optimized for short sequences) (Supplementary File 5). The dissimilarity between each of the predicted peptides and the predicted proteomes of the following organisms was also calculated and used to remove candidate targets with high similarity scores, which could result in diagnostics with a higher rate of false positives:

- *Homo sapiens* (GCF 000001405.40).
- *Sarcoptes scabiei* (GCA 000828355.1), Measles morbillivirus (GCF 000854845.1), and *Treponema pallidum* (GCF 000246755.1), pathogens with potentially similar clinical presentations [4].
- All sequences available from NCBI under family Poxviridae and not identified as Monkeypox (a total of 576 isolates of 72 species).

All resulting BLASTp files and identifiers of all sequences are provided in Supplementary File 6.

Figure 2 illustrates the results of this filtering step. Of the 66 unique peptides preselected based on the probability score attributed by the model and their similarity score to known LBCE extracted from the IEDB, 9 displayed dissimilarities to human and poxvirus proteins above our cutoff thresholds (≥0.5 dissimilarity from human proteins; ≥0.05 dissimilarity from proteins from other Poxvirus), in addition to high conservation scores (≥0.75 similarity for the 88 MPXV isolates).

We selected these nine peptides predicted as containing LBCEs, highlighted as green triangles in Fig. 2, for further experimental evaluation. These peptides are found in proteins fulfilling a variety of roles in MPXV biology and encompassing both early and postreplicative expressed genes. These roles range from virulence factors secreted by infected cells that inhibit molecular hubs of the host immune response to structural components of viral particles and enzymes playing roles in viral DNA replication (Supplementary File 8).

### Predicted linear B-cell epitope–containing regions are recognized by IgG and IgM from naturally orthopoxvirus-infected individuals

**Figure 3:**
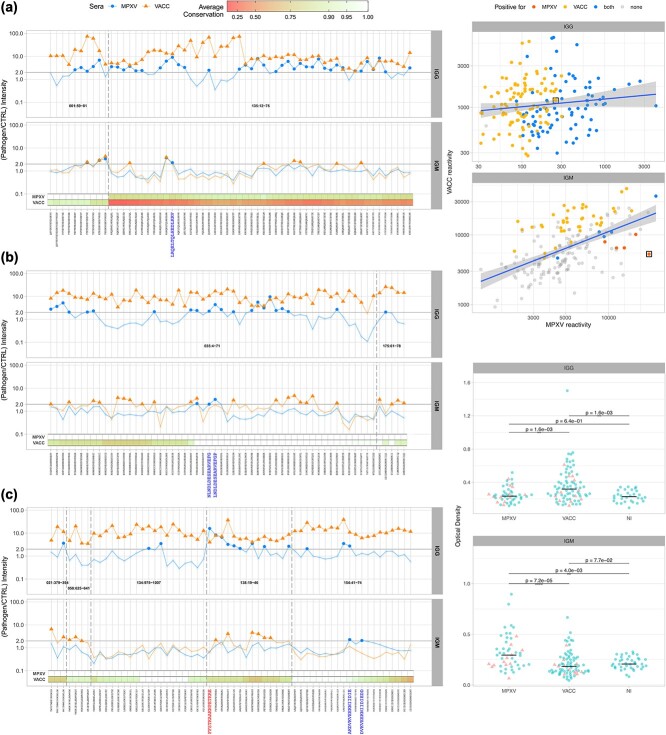
Experimental validation of predicted targets. (a) Immunoblotting results as IgG and IgM reactivity ratios (MPXV/Control and VACV/Control) of the sera pools against the 179 peptides extracted from the nine epitope-containing regions predicted by the phylogeny-aware pipeline. Peptide/sera pairs for which the observed intensity ratio was lower than 2 are shaded to highlight the more reactive ones. The color bars represent the within-clade conservation profile of each peptide for MPXV and VACC. Six peptides are highlighted as potential targets that could be used to indicate recent MPXV infections (MPXV/control >2 and VACV/control <2). Five of these are new discoveries, while the peptide FFSTKAAKNPETKRE is an independent rediscovery of the one reported in [33] as a potential target for differential serodiagnosis of MPXV. (b) Correlation between the seroreactivity of the peptides to the sera of patients infected with VACC versus MPXV. IgG (top panel, Pearson *r* = 0.035, *P*-value = .63); IgM (bottom panel, Pearson *r* = 0.527, *P*-value = 3 × 10^−14^). Each point corresponds to a different peptide. Seroreactivity values above the cutoff for only MPXV, only VACC, both, or none are indicated in the figure. The peptide DVKVEEKNIIDIEDD is highlighted in both panels with a black box. (c) ELISA results for peptide DVKVEEKNIIDIEDD using individual patient sera. Each dot corresponds to a different patient. Points shown as triangles represent sera from patients that composed the pools in the immunoblotting screening. Notice that the ELISA results support the immunoblotting results for the peptide (higher VACC IgG and higher MPXV IgM reactivity). Results marked as “NI” correspond to controls (“not infected”).

To validate the LBCE predictions, we synthesized 179 peptides of 15 amino acids covering the full extent of the nine LBCE-containing regions in a immunoblot membrane and evaluated their reactivity to IgG and IgM. For that, we initially used pools of sera from patients infected with MPXV or VACV, as well as healthy donors, to perform an initial screening of the putative LBCE-containing regions for possible epitopes (Fig. 3a). We computed sequence conservation information for the MPXV and VACV isolates available as of June 2022 (88 and 68 isolates, respectively). Overall, the sequences are more conserved in MPXV, with all but one peptide conserved in 100% of isolates.

We defined all peptides where reactivity for either MPXV-infected or VACV-infected were at least two times greater than the reactivity for the pool of healthy donors as positives. All nine LBCE-containing regions had at least one peptide reactive to IgG pools of sera from VACV patients (eight in the case of MPXV) when compared with healthy donors (Fig. 3a). At the individual peptide level, we observed that all 179 (100%) were reactive to the VACV-IgG sera pool, while 79 (44%) were reactive to MPXV-IgG patient sera (Fig. 3a and b). This stronger peptide reaction profile in VACV-infected individuals when compared to MPXV-infected individuals is likely due to the scenario of recurring infections of workers of the dairy industry, which comprises a considerable fraction of our VACV cohort [34].

Four of the peptides have the IgG intensity for the MPXV sera pool higher than the VACV samples. Interestingly, the one with the highest MPXV/VACV reactivity ratio (3.97, identified in Supplementary Table ST5 as 138:19-46; Fig. 3a, highlighted in red) coincides with a peptide previously reported as a highly specific MPXV epitope [33] (*te***FFSTKAAKNPETKRE**AIVKAYGDDNEET*lkq*; uppercase characters highlighting our prediction and bold highlighting the specific peptide that returned the highest MPXV/VACV reactivity ratio). In agreement with our results, this epitope is recognized by a monoclonal antibody of the IgG1 subclass [35]. The detection of this epitope, which was at no point part of the training set (as it is not present in the IEDB export used to train our models), corroborates both the ability of the selected model to accurately detect LBCE and the usefulness of the predictive pipeline developed in this work for identifying valuable targets of diagnostic potential (Supplementary Table ST2).

A total of eight and four, respectively, out of the nine LBCE-containing regions evaluated had at least one peptide reactive to IgM from patients with VACV or MPXV (Fig. 3a). At the individual peptide level, 44 (25%) and 10 (6%) were reactive to VACV-IgM or MPXV-IgM sera, respectively, with 5 of those (3%) being reactive to both pools of sera. The IgM reactivity of MPXV- and VACV-infected individuals had a stronger correlation (Pearson *r* = 0.527, *P*-value = 3 × 10^−14^) than the IgG one (Pearson *r* = 0.035, *P*-value = .63; see Fig. 3b). Even though we did not further investigate this issue, we argue that it is likely caused by the distinctive nature of the cohorts (early MPXV infection versus ancient and recurring VACV infection), which is likely to increase both the IgG and IgM responses in the VACV cohort and only the IgM response in the MPXV samples.

Five peptides were only reactive to MPXV-IgM (two in target 035:4-71, two in 154:41-74, and one in 135:12-75; highlighted in blue in Fig. 3a). Four of these were 100% conserved across the 88 MPXV isolates, with the fifth presenting a conservation score of 83.5%. Four of these present conservation scores above 99% (between 94.2% and 99.8%) across the 68 VACV isolates, with the remaining one having a conservation score for VACV estimated as 33.1%. Of these five peptides, the proportional difference in IgM reactivity for VACV and MPXV sera pools of one of the peptides extracted from target 154:41-74 (peptide DVKVEEKNIIDIEDD, MPXV/VACV reactivity ratio = 3.60) is substantially greater than that of the other four. Furthermore, when considering the IgG profile, the opposite profile is observed, as it is only reactive for the VACV sera pool.

We synthesized this peptide and evaluated its reactivity of our cohort through ELISA, using individual sera from MPXV- and VACV-infected patients (Fig. 3c). Our results supported the previous immunoblotting results, showing that the average IgM reactivity of sera from MPXV-infected individuals was significantly higher than either sera from VACV-infected individuals or healthy donors (Fig. 3c, “IGM”); Wilcoxon rank-sum *P*-values of 7.2 × 10^−5^ and 4.0 × 10^−3^, respectively), while sera from VACV-infected individuals do not significantly differ from healthy donors (Wilcoxon rank-sum *P*-value = 7.7 × 10^−2^).

In agreement with the immunoblotting results, the average IgG reactivity of sera from VACV-infected individuals was significantly higher than either MPXV-infected patient sera samples or healthy donors (Fig. 3c, “IGG”; Wilcoxon rank-sum *P*-value = 1.6 × 10^−3^ for both tests). We found no significant difference in average IgG reactivity between MPXV-infected sera and healthy donors (Wilcoxon rank-sum *P*-value = .64).

## Discussion

In data science and applied machine learning, a major conceptual step is domain understanding. In general terms, this means understanding specific properties and caveats of data emerging from a specific data-generating process to account for eventual biases that may interfere in downstream analyses. In biology, data related to different species are nonindependent due to common ancestry [36]. This fact alone generates two major consequences: the first one is that any strategy that uses species-centric data to draw conclusions, such as genotype–phenotype associations studies of species data, must consider this nonindependence to prevent overestimation of model performance [37,38]. On the other hand, the fact that phylogenetically closer species are expected to be more similar in both their phenotypes and genotypes can be leveraged to produce tailored, phylogeny-aware statistical models for specific groups of species with superior performance compared against generalist approaches. By training ML models using data-rich species, these can also be successfully applied to make predictions for data-poor, phylogenetically close organisms.

This rationale has been widely adopted in several fields of computational biology, even though it is not always explicitly stated. Algorithms for gene prediction in eukaryotic genomes, for instance, are usually trained using data from many phylogenetically distant species that represent major eukaryotic groups [39]. This approach produces multiple models sharing an overall statistics scaffold but with individual models tailored for a major group of organisms. The motivation of this strategy is that phylogenetically closer species will have shared genomic properties that are useful to predict gene model structures, such as genome nucleotide composition, dinucleotide and codon usage, and intron and exon lengths.

In the context of LBCE prediction, as we demonstrated in this study, a similar strategy can leverage the nonindependence of pathogen data to train models for specific groups of pathogens using data from related species that can be informative for predicting epitopes in data-poor organisms, such as neglected diseases or emerging pathogens. By combining a predictor built under a phylogeny-aware framework with sequence conservation and similarity analyses, we developed an optimized pipeline for the prediction and prioritization of LBCE targets in OPVs. Using this pipeline, we selected nine potential LBCE-containing regions. One of those was an independent rediscovery of a previously described peptide from protein A29L [33] (which appears in the present study under the ID MPXV-USA 2022 MA001-138.t01) as a promising target for the specific diagnostic of MPXV. We highlight that this peptide was not present in the training set of our models, as it is not contained in the IEDB export used at the time of model development.

The differential serodiagnosis of OPV infection, as well as the distinction between infection and vaccinated individuals, is challenging due to the lifelong production of antibodies against these pathogens and their considerable antigenic and serologic cross-reactivity [40, 41]. We have also identified a novel epitope with the potential to detect recent MPXV infections based on IgM sera reactivity. A recent study outlined the development of a highly sensitive and specific diagnostic test capable of distinguishing previous MPXV infection from vaccination status through the analysis of IgG profiles [30]. While our research did not specifically aim to fully develop an immunodiagnostic test, this recent advancement toward that end provides valuable context for our findings. The reported high performance was achieved through the utilization of a combination of MPXV-specific peptides and cross-reactive OPV proteins, underscoring the challenging nature of distinguishing between different OPVs. Moreover, their method primarily focuses on evaluating IgG profiles, which would be limited to the detection of subclinical MPXV infections and to providing an epidemiological overview of MPXV spread within communities. Our findings suggest the feasibility of developing a diagnostic kit akin to the ensemble of proteins and peptides described by Yates *et al*. [30] to differentiate between recent and past infections of MPXV and VACV, as well as vaccinated individuals, based on IgM reactivity for this peptide.

Beyond the detection of potential Monkeypox LBCEs, the phylogeny-aware approach used here has been shown to provide equivalent or superior results than current standard tools for LBCE prediction, reinforcing earlier results [10]. More importantly, the successful development of a model tailored specifically for an emerging global pathogen with almost no LBCE data available shows that this approach is useful even beyond the lower bounds investigated in earlier work [22]. The incorporation of information from phylogenetically related pathogens can be a successful strategy for the development of predictive models tailored for specific groups of pathogens that share a common ancestor. With the current wide availability of considerable computing power, there is scope for a wider use of pathogen-specific models, trained and tuned specifically for the prediction task of interest.

In agreement with our modelling strategy, recent developments in LBCE prediction include the integration of taxonomic data into predictive models, highlighting the potential to capitalize on distinct epitope signatures among pathogen groups [16, 17, 42–45]. It should be clear, however, that the innovative aspect of our work is not in the feature space used or in the deployment of complex predictive models. On the contrary, our results indicate that it is possible to achieve results that are comparable to, if not better than, the state-of-art tools, despite using a much smaller and computationally simpler feature space combined with a traditional data mining workflow. The most innovative aspect of our work relates instead to the curation of training data based on evolutionary relationships between the source organisms of known epitopes/nonepitope peptides and the target pathogen. Indeed, using more sophisticated modeling approaches and feature spaces, such as the ones used by current state-of-the-art generalist predictors, in combination with the phylogeny-aware selection of training data presented in this work, would potentially yield even better results. Challenges persist, however, in determining the ideal taxonomic levels for model training and ensuring methodologically rigorous performance evaluations. Addressing these issues is essential for realizing the full potential of phylogeny-aware epitope prediction in practical applications within biomedical research and development.

**Key Points**

- Description of a phylogeny-aware framework for training pathogen-specific linear B-cell epitope predictors.
- Development of a predictor tailored for orthopoxviruses, including monkeypox and vaccinia.
- Experimental validation reveals new epitopes in individuals exposed to monkeypox and vaccinia viruses.

## Supplementary Material

Supplementary_Files_bbae527_2

Supplementary_tables_bbae527_2

## Data Availability

The data underlying this article are available in Zenodo, at https://doi.org/10.5281/zenodo.7838331.
